# Information Needs and Fear of Radiotherapy in Women With Breast Cancer

**DOI:** 10.7759/cureus.88540

**Published:** 2025-07-22

**Authors:** Apostolina Ouzouni, Georgios A Plataniotis, Antonio Capizzello, Areti Gkantaifi, Areti Tsaloglidou, Antonios Galanos, Maria Lavdaniti

**Affiliations:** 1 Department of Clinical Pathology, American Hellenic Educational and Progressive Association (AHEPA) Hospital, Aristoteles of Thessaloniki, Thessaloniki, GRC; 2 Department of Radiation Oncology, American Hellenic Educational and Progressive Association (AHEPA) Hospital, Aristoteles of Thessaloniki, Thessaloniki, GRC; 3 Department of Nursing, International Hellenic University, Thessaloniki, GRC; 4 Laboratory for Research of the Musculoskeletal System, KAT General Hospital, School of Medicine, National and Kapodistrian University of Athens, Athens, GRC

**Keywords:** breast cancer, educational intervention, informational needs, patient fear, psychological distress, radiotherapy

## Abstract

Introduction: Radiotherapy constitutes a fundamental modality in breast cancer treatment; however, it is often accompanied by considerable patient fear, psychological distress, and unmet informational needs. Addressing these factors may enhance patient compliance and quality of care.

Methods: A longitudinal cohort study was conducted at two radiotherapy departments in Thessaloniki, Greece, from October 2022 to May 2023. A total of 216 breast cancer patients were enrolled and assigned to a control group (n = 98), receiving standard care, or an intervention group (n = 118), receiving an informational brochure detailing radiotherapy procedures. Validated Greek versions of the Questionnaire for Assessing Fear of Radiotherapy in Oncology Patients (QAFRT), the M.D. Anderson Symptom Inventory (MDASI), the Information Styles Questionnaire (ISQ), and the Profile of Mood States Short Form (POMS-S) were administered at baseline and upon completion of radiotherapy.

Results: At baseline, groups were homogeneous in demographics and clinical characteristics, except for education level (P < 0.0005). Significant reductions in fear scores were observed in the intervention group across all QAFRT subscales (P < 0.0005), including fear of treatment effectiveness, illness during therapy, daily life disruption, and side effects. The intervention group also demonstrated significant improvements in psychological well-being, with reductions in tension, depression, anger, fatigue, and confusion, and increased vigor on the POMS-S (P < 0.0005). Symptom burden, as assessed by MDASI, improved significantly across all domains in the intervention group (P < 0.0005). Furthermore, informational needs regarding disease and treatment were significantly reduced in the intervention group post-intervention (P = 0.001), while psychological informational needs remained unchanged.

Conclusion: Providing structured, written informational support prior to radiotherapy initiation significantly reduces fear, improves mood states, and alleviates symptom burden in breast cancer patients. These findings highlight the importance of tailored patient education in optimizing radiotherapy experiences and psychological outcomes.

## Introduction

Cancer is the second leading cause of death worldwide. As reported by the Global Cancer Agency, there were around 19,976,499 new cancer cases in 2022, resulting in 9,743,832 fatalities. As one of the most prevalent chronic illnesses, cancer exhibits features such as high rates of illness, mortality, and recurrence. Concurrently, advancements in medical technology have led to an increased survival rate among patients with solid tumors [1]. Many cancer patients undergo a comprehensive treatment approach, which includes surgery, radiotherapy, chemotherapy, targeted therapy, and immunotherapy [2].

Radiotherapy is recommended for about 50% of cancer cases globally, leading to significant enhancements in local tumor management and overall survival rates for patients with various types of cancer across multiple disease locations [2]. Radiotherapy can cause various adverse effects, including skin reactions, oral complications, and fatigue, which can amplify patients’ physical burden and significantly escalate their psychological stress. As a result, numerous patients undergoing radiotherapy often face negative emotions such as anxiety, worry, and fear. Fear is characterized as an unpleasant and often intense emotion triggered by the expectation or realization of danger, accompanied by heightened autonomic activity. Furthermore, fear has been consistently recognized in research as a prevalent issue with detrimental psychological and functional effects [3].

As noted by Arikan et al., patients with cancer who receive radiation therapy may experience anxiety and depression due to fears surrounding the radiotherapy machines, concerns about potential negative outcomes during treatment, and doubts about the effectiveness of the radiation. Furthermore, the research revealed that 27.1% of patients reported that their anxiety was linked to the fear of being unable to handle the side effects of radiotherapy [4].

The findings from the research conducted by Shaverdian et al. revealed that nearly 50% of the patients indicated they had previously encountered alarming stories regarding the serious side effects of radiation. The most frequently mentioned initial concerns about radiotherapy included harm to internal organs, skin burns, the fear of becoming radioactive, and the inability to carry out daily activities. Other worries included fatigue and weakness, harm to the immune system, pain, alterations in appearance, and expenses. Only around 6% of patients expressed that they felt no fear regarding radiotherapy. Furthermore, this research highlighted the prevalence of fears and misconceptions surrounding breast radiotherapy and demonstrated that patients' actual experiences with treatment tend to surpass their earlier expectations. It is noteworthy that a significant majority of patients concurred that their initial negative feelings and concerns about breast radiotherapy were unjustified [5].

There is limited understanding of the information-seeking behaviors, needs, and perceptions of breast cancer patients regarding breast radiotherapy before they consult with a radiation oncologist. Murchison et al. evaluated these aspects to identify possible gaps and overlaps in the educational process related to breast radiotherapy. The primary sources of information about breast cancer radiotherapy cited were healthcare professionals (55%), friends or family members who had undergone radiotherapy (53%), and online sources (45%). A significant majority (79%) had minimal or no understanding of radiotherapy. Sixty-seven percent expressed a little to moderate level of concern about breast radiotherapy, while 29% reported being very concerned. Half of the respondents were uncertain about the advantages of breast radiotherapy, and 46% believed it would offer a moderate to considerable benefit. Therefore, a deeper understanding of how patients are educating themselves and the accuracy of the information they obtain can assist physicians in addressing their concerns and guiding their choices [6].

Most research continues to focus on the immediate and short-term effects of cancer, primarily at diagnosis, during treatment, cancer recurrence, or during the first years after treatment [3-5]. Research has shown that there are only a limited number of studies regarding cancer patients' fears of radiation and their informational requirements.

Consequently, the purpose of this study was to assess breast cancer patients’ fear of radiotherapy and their information needs for the radiation oncology consultation. To the best of our knowledge, in Greece, there have been no similar studies, except from Ouzouni et al., who conducted a cross-sectional pilot study to validate the Greek translation of the Questionnaire for Assessing Fear of Radiotherapy in Oncology Patients (QAFRT) [7].

## Materials and methods

Study design and participants

This study is a longitudinal cohort conducted in two radiotherapy departments: the first in a Greek university hospital and the second in an anticancer hospital in Thessaloniki.

The estimation of sample size was performed using the statistical program G*Power 3.1 [8], with the type 1 error probability (α error probability) set at 0.05, a moderate effect size, and a power factor (1-β error probability) of 0.90. For two groups and four measurements, the program estimated a minimum sample size of 108 patients. The sample consisted of patients with histopathologically confirmed breast cancer, attending the oncology outpatient department (outpatients) or being hospitalized (inpatients), from October 2022 to May 2023. Five cancer patients refused to answer the questionnaire.

The criteria for inclusion in the study were as follows: a histologically confirmed breast cancer diagnosis, receiving radiation treatment, being at least 18 years old, being able to communicate verbally and fluently in both written and spoken Greek, and providing consent to participate in the study. Alcohol and/or drug misuse, dementia, brain metastases, a history of psychotic illness, and a lack of diagnosis knowledge were the exclusion criteria.

Participants were separated into two groups. The control group consisted of 98 patients, and the intervention group consisted of 118 patients. Participants in the control group completed the questionnaires during the first five radiotherapy sessions (baseline), followed by a final assessment during the last five radiotherapy sessions (final measure). Participants in the intervention group completed the questionnaires during the first five radiotherapy sessions (baseline), then received an informational brochure leaflet containing detailed information about radiotherapy, and were assessed again during the last five radiotherapy sessions (final measure).

Measures

Demographic data, diagnosis, and clinical characteristics were obtained from patients' medical records. All tools and scales used in the study are freely available; however, formal permission for their use was obtained from the original authors, as required.

The Greek version of the M.D. Anderson Symptom Inventory (MDASI) allows a brief measurement of symptoms related to cancer and its treatment. The MDASI consists of 13 symptoms that are rated based on their presence and severity. Each symptom is rated on an 11-point scale (0-10) to indicate its presence and severity. Specifically, 0 means “not present” and 10 means “as bad as you can imagine,” referring to the last 24 hours. It also includes six additional questions related to the impact of symptoms on daily life and activities, with responses on a 10-point scale (0 = the symptom did not interfere to 10 = the symptom interfered completely). This specific questionnaire has been validated in the Greek population (Cronbach's α = 0.8466) [9].

The Greek version of the Information Styles Questionnaire (ISQ) is a specific tool designed to measure the information needs of cancer patients. It was created in 1980 [10] and has been found to have validity and reliability [11]. It consists of three parts and a total of 29 questions. Specifically, the first part measures the amount of information that patients wish to receive, on a five-point Likert scale (1 = no more information than necessary to 5 = as much information as possible). The second part assesses the desire for specific types of information about diseases, treatment options, and psychosocial needs, through three possible answers (“It is absolutely necessary for me to know this information,” “I would like to know this information,” “I do not want this information”). Finally, in the third part, patients are asked to choose a statement that best describes their general attitude toward information about their illness (“I want to know only the information that is necessary to be able to take good care of myself,” “I want to get more information, but only if it is pleasant,” “I want as much information as possible, whether it is pleasant or unpleasant”). After factor analysis, two factors were revealed. The first factor, “Disease and Treatment,” had a Cronbach's α = 0.92, and the second factor, “Psychological,” had a Cronbach's α = 0.89. In Greece, the tool was evaluated by Alamanou et al. [12].

The Greek version of the Profile of Mood States-Short Form (POMS-S) [13] was created to assess individual mood. The full version of the questionnaire includes 60 statements and was created in 1971. The short form, created in 1992 by the same researchers, includes 30 statements. It contains six subscales: tension-anxiety, anger-hostility, sadness, fatigue, confusion, and vitality. Each item presents an affective adjective, and participants are asked whether the adjective corresponds to how they have felt during the past week. There is a five-point scale from 0 = “not at all” to 4 = “very much.” The values are then summed, with higher scores indicating worse psychological mood. This specific scale was translated and adapted into Greek by Roussi and Vassilaki, and the internal validity reliability of the subscales ranges from 0.69 to 0.91 (Cronbach’s α = 0.91) [14].

The Questionnaire for Assessing Fear of Radiotherapy in Oncology Patients [15] is used to assess the level of fear of radiotherapy in oncology patients. It consists of 15 questions, each with five possible answers on a Likert scale. The translation and validation of the questionnaire in the Greek population were conducted by Ouzouni et al. [7], and the reliability was confirmed with Cronbach’s α = 0.82. Exploratory factor analysis performed on the translated version resulted in a refined 13-item questionnaire encompassing four factors: (1) fear of radiotherapy effectiveness, (2) fear of illness during radiotherapy, (3) fear of radiotherapy's impact on daily life, and (4) fear of side effects and relationships. Patients with high total scores may require special attention and adequate support to reduce adverse effects and ensure full and adequate implementation of treatment.

Patients who feel unsatisfied with the information provided may act non-compliantly and require more time compared to those who receive the necessary information [16]. Based on previous survey findings [16], we developed an informational brochure leaflet that includes all the appropriate information patients need before, during, and after radiotherapy. Patients primarily preferred written information, as it allowed them to meet their specific information needs as they arose [16]. They took the printed informational brochure leaflet home and could refer to it whenever needed, which may significantly improve patient understanding of how radiotherapy works.

Ethical considerations

Written permissions from the creators of the questionnaires were obtained by the research team for the use of the above-mentioned questionnaires. Ethical approval to conduct the study was granted by the scientific committee of the participating hospitals (approval number: 10035/23.02.2022), as well as by the Ethics Committee of the International Hellenic University of Thessaloniki (approval number: 14/01.11.2022). Before completing the questionnaire, patients were provided with the necessary information and signed a consent form. The confidentiality of the gathered data was maintained in accordance with Regulation (EU) 2016/679 of the European Parliament concerning the protection of individuals with regard to the processing of personal data and the free movement of such data, repealing Directive 95/46/EC (General Data Protection Regulation).

Statistical analysis

Data were expressed as mean ± SD, and the Kolmogorov-Smirnov test was used to examine the normal distribution of the parameters.

The comparison for homogeneity between intervention groups with respect to demographic and clinical variables was performed using the independent samples t-test and Fisher’s exact test.

We used the two-way mixed ANOVA model, with “intervention” as the between-group factor and “time” as the within-group factor, to analyze measurements, applying the Bonferroni correction for all pairwise comparisons either between or within groups.

Sensitivity analysis of variables concerning baseline balance between groups was performed using an analysis of covariance model (ANCOVA), with the absolute change from the initial to the final value of the intervention as the dependent variable, the group (control or intervention) as the factor, and the initial values of the measures as the covariate.

All tests were two-sided, and statistical significance was set at p < 0.05. All analyses were carried out using IBM SPSS Statistics for Windows, Version 21 (Released 2012; IBM Corp., Armonk, New York).

## Results

Homogeneity between the compared groups

Patients’ demographic profiles and clinical characteristics are presented in Table 1. The mean age of both group members was close to 60 years. The majority of patients in both groups had breast cancer on the left side of the body. Most women in both groups underwent surgery and chemotherapy. Moreover, 78 patients from the control group and 84 patients from the intervention group had concomitant diseases.

**Table 1:** Homogeneity between the compared groups

Variable		Control Group (n = 98)	Intervention Group (n = 118)	p-value
Age (mean ± SD)		60.12 ± 10.44	59.43 ± 9.74	0.616
Marital status	Single	12 (12.2%)	16 (13.6%)	0.841
	Married	86 (87.8%)	102 (86.4%)	
Education	Primary	45 (45.9%)	46 (39.0%)	<0.0005
	Secondary	26 (26.5%)	66 (55.9%)	
	Tertiary (MSc-PhD)	27 (27.5%)	6 (5.0%)	
Breast cancer	Right	47 (48.0%)	58 (49.2%)	0.892
	Left	51 (52.0%)	60 (50.8%)	
Metastasis	No	97 (99.0%)	112 (95.0%)	0.130
	Yes	1 (1.0%)	6 (5.0%)	
Surgery	No	0 (0.0%)	4 (3.4%)	0.128
	Yes	98 (100.0%)	114 (96.6%)	
Comorbidities	No	20 (20.4%)	34 (28.8%)	0.207
	Yes	78 (79.6%)	84 (71.2%)	
Chemotherapy	No	11 (11.2%)	21 (17.8%)	0.107
	Yes	87 (88.8%)	97 (82.2%)	
Hormone therapy	No	94 (96.0%)	106 (90.0%)	0.118
	Yes	4 (4.1%)	12 (10.0%)	
Travel from another city for radiotherapy	No	61 (62.2%)	67 (58.8%)	0.487
	Yes	37 (37.8%)	51 (43.2%)	
Number of radiotherapy sessions before the intervention	Median (Min–Max)	3.0 (1–5)	2.5 (1–5)	0.210
Number of radiotherapy sessions after the intervention	Median (Min–Max)	18 (11–24)	19 (5–30)	0.250

There is no statistically significant difference between the groups for all demographic and clinical characteristics except for education level (p<0.0005).

Fear questionnaire

According to the mixed model ANOVA for the variable “Fear,” there was a statistically significant interaction between the factors “group” and “time,” F(1, 214) = 136.83, p < 0.0005 (Table 2).

**Table 2:** Mixed model ANOVA by two factors for the variable "Fear" SD: standard deviation, SE: standard error, BG: between group, WG: within group.

Variables	Group	Baseline	Final	p-value_WG_	Absolute Change Baseline-Final
		Mean ± SD	Mean ± SD		Adjusted Mean ± SE
Fear total	Control	26.61±4.79	32.58±5.48	<0.0005	7.88±0.69
	Intervention	23.75±8.76	47.53±7.95	<0.0005	22.19±0.63
	p-value_BG_	0.004	<0.0005		<0.0005
Fear Factor 1	Control	7.88±1.84	9.48±2.11	<0.0005	1.78.±0.25
	Intervention	7.49±2.67	13.14±2.79	<0.0005	5.49±0.23
	p-value_BG_	0.227	<0.0005		<0.0005
Fear Factor 2	Control	2.68±0.93	3.51±1.03	<0.0005	0.90±0.13
	Intervention	2.52±1.41	5.54±1.44	<0.0005	2.96±0.12
	p-value_BG_	0.317	<0.0005		<0.0005
Fear Factor 3	Control	4.02±1.51	4.88±1.56	<0.0005	1.15±0.17
	Intervention	3.33±2.31	7.45±1.83	<0.0005	3.87±0.15
	p-value_BG_	0.012	<0.0005		<0.0005
Fear Factor 4	Control	8.03±1.88	9.57±1.98	<0.0005	2.54±0.21
	Intervention	6.47±2.96	14.00±2.51	<0.0005	6.70±0.23
	p-value_BG_	<0.0005	<0.0005		<0.0005

There was a statistically significant difference in the “Fear” index from the baseline to the end of radiotherapy due to the intervention in the intervention group (p < 0.0005).

There was also a statistically significant difference between the groups at baseline (p = 0.004) and at the end of radiotherapy (p < 0.0005).

There was a statistically significant difference in the absolute change from baseline to the end of radiotherapy in the “Fear” index, adjusted for the baseline value, between the groups (p < 0.0005). The intervention group, because of the informational brochure leaflet, showed a reduced level of fear.

Furthermore, in all four factors of the questionnaire (Factor 1: fear of radiotherapy effectiveness, Factor 2: fear of illness during radiotherapy, Factor 3: fear of radiotherapy's impact on daily life, and Factor 4: fear of side effects and relationships), there was a statistically significant difference between the groups at the end of radiotherapy (p < 0.0005).

POMS questionnaire

Comparisons of the absolute values of the POMS questionnaire variables (Tension, Depression, Anger, Fatigue, Vigor, and Confusion) between the two measurements showed a statistically significant difference from baseline to the end of radiotherapy (Table 3).

**Table 3:** Mixed-model ANOVA by two factors for the POMS questionnaire variables POMS: Profile of Mood States.

Variable	Group	Baseline	Final	P-Value Within Group	Absolute Change
Tension	Control	12.74±1.82	11.59±1.80	p<0.0005	-1.63±0.27
	Intervention	13.88±3.41	9.22±3.24	p<0.0005	-4.26±0.24
Depression	Control	14.02±2.47	12.21±2.83	p<0.0005	-1.62±0.37
	Intervention	13.02±4.45	8.63±5.33	p<0.0005	-4.55±0.34
Anger	Control	8.14±1.67	7.13±1.90	p<0.0005	-1.38±0.23
	Intervention	9.34±2.43	6.13±2.73	p<0.0005	-2.91±0.21
Fatigue	Control	9.69±1.69	8.57±1.38	p<0.0005	0.74±0.18
	Intervention	9.03±1.40	7.35±2.05	p<0.0005	-2.11±0.16
Vigor	Control	6.81±1.66	8.00±1.97	p<0.0005	1.39±0.21
	Intervention	5.94±2.43	8.38±2.70	p<0.0005	2.28±0.19
Confusion	Control	7.00±2.22	6.27±2.12	p<0.0005	-0.96±0.23
	Intervention	7.86±2.45	5.09±2.87	p<0.0005	-2.58±0.21

More specifically, there was a statistically significant difference in the Tension variable from baseline to the end of radiotherapy for both the control group (p < 0.0005) and the intervention group (p < 0.0005) (Table 3).

There was also a statistically significant difference between the groups at baseline and at the end of radiotherapy (Table 3).

There was a statistically significant difference in the absolute change from baseline to the end of radiotherapy in the Tension variable of the POMS questionnaire, adjusted for the baseline value, between the groups (p < 0.0005). The intervention group reduced the levels of tension (−4.26 ± 0.24) by the end of radiotherapy (Table 3).

Similarly, there was a statistically significant difference in the absolute change from baseline to the end of radiotherapy in the Depression, Anger, Fatigue, Vigor, and Confusion variables, adjusted for the baseline values, between the groups (p < 0.0005) (Table 3). Specifically, participants in the intervention group reduced the levels of depression (−4.55 ± 0.34), anger (−2.91 ± 0.21), fatigue (−2.11 ± 0.16), and confusion (−2.58 ± 0.21), and improved the level of vigor (2.28 ± 0.19) at the end of radiotherapy.

MDASI questionnaire

According to Mystakidou et al. [9], in the Greek translation and validation of the M.D. Anderson Symptom Inventory (MDASI), factor analysis resulted in a three-factor model. Specifically, Factor 1 (Health Orientation Care) included enjoyment of life, walking, relationship with people, general activity, sadness, and pain; Factor 2 (Job/Household Duties) included dry mouth, numbness or tingling, loss of memory, nausea, constipation, and emesis; and Factor 3 (Relationships with Family) included distress, shortness of breath, sleep disturbance, loss of appetite, fatigue, and sleepiness.

**Figure 1:**
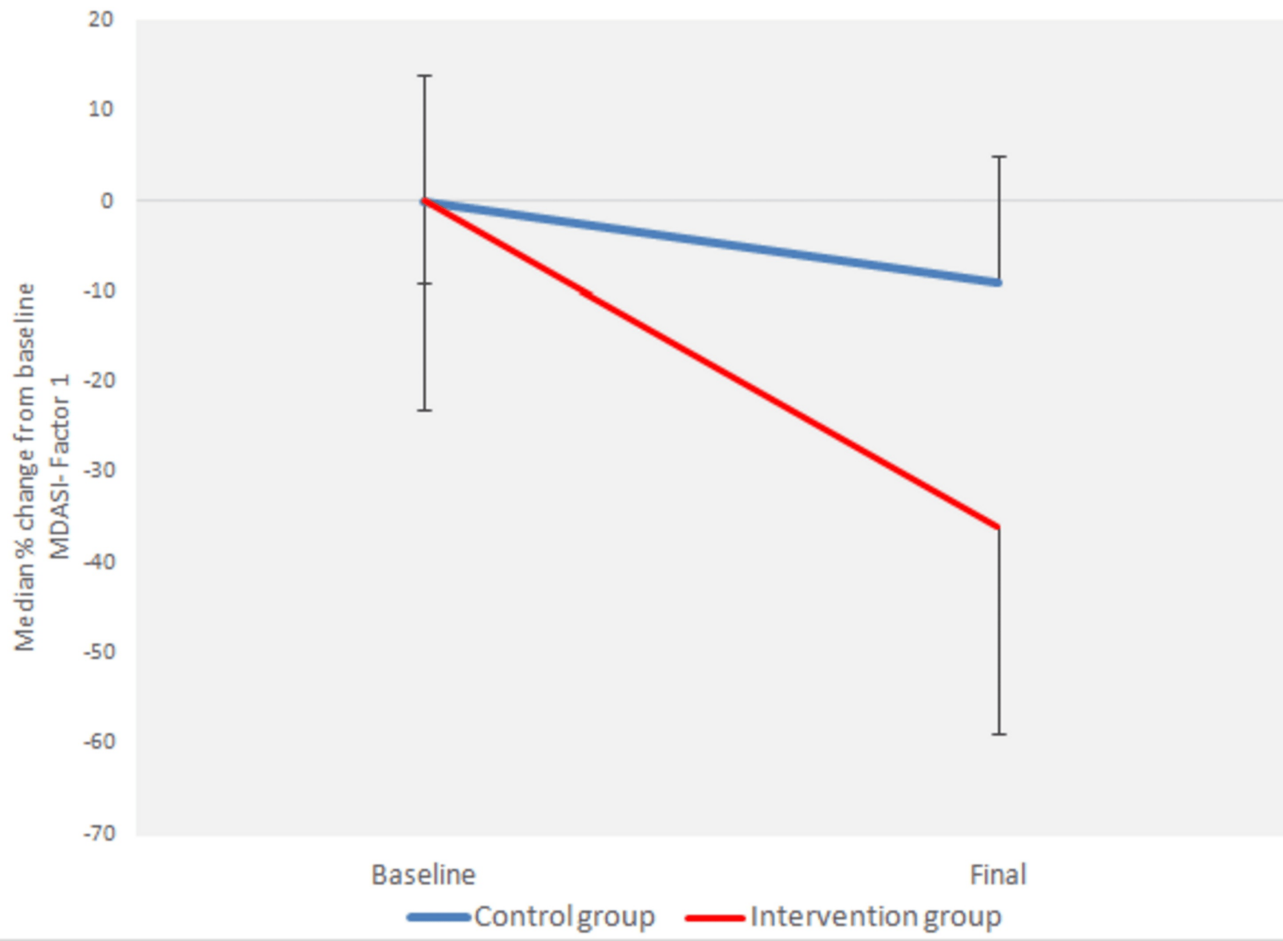
Median percentage change from baseline of MDASI Questionnaire - Factor 1 MDASI: M.D. Anderson Symptom Inventory.

**Figure 2:**
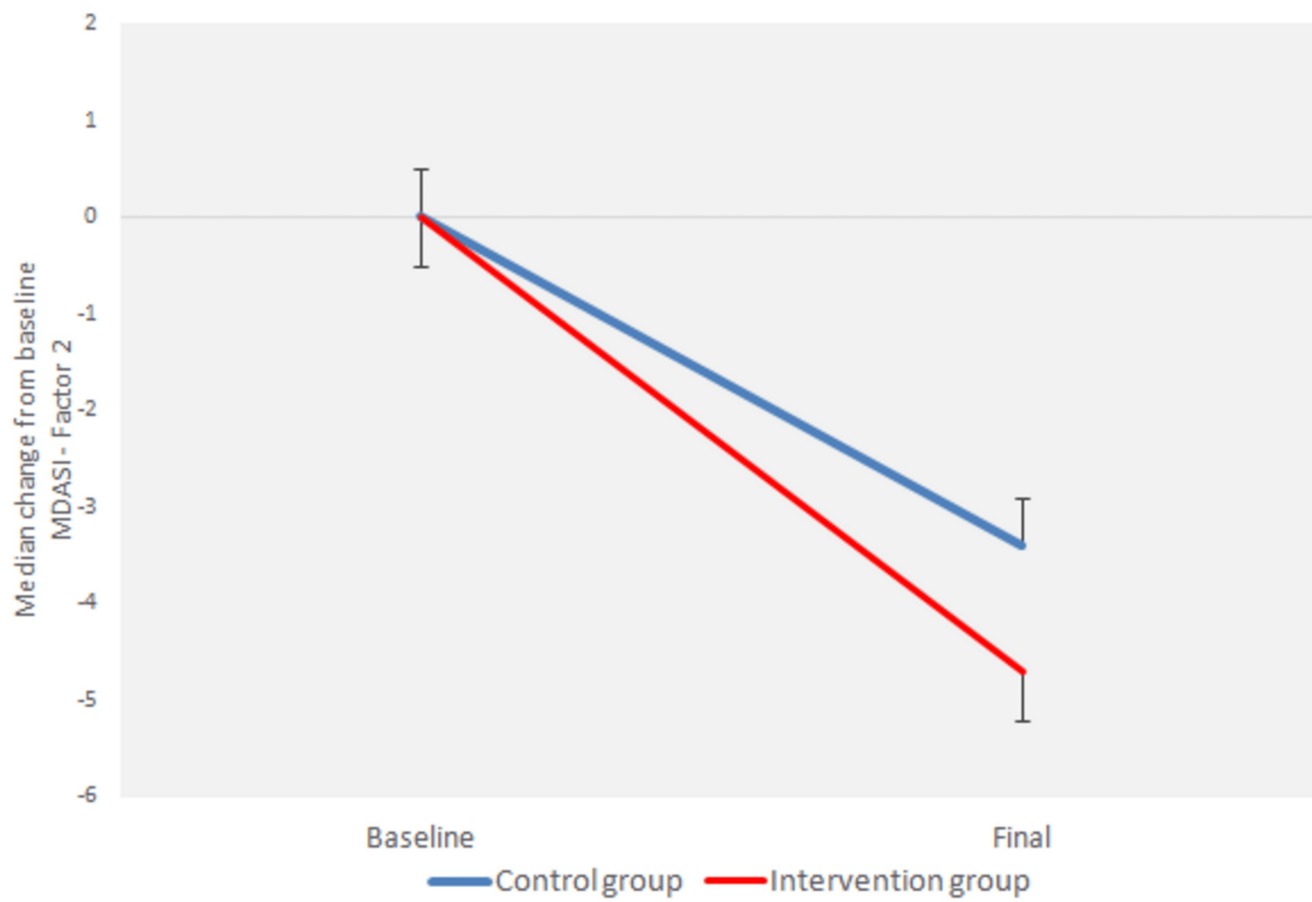
Median percentage change from baseline of MDASI Questionnaire - Factor 2 MDASI: M.D. Anderson Symptom Inventory.

**Figure 3:**
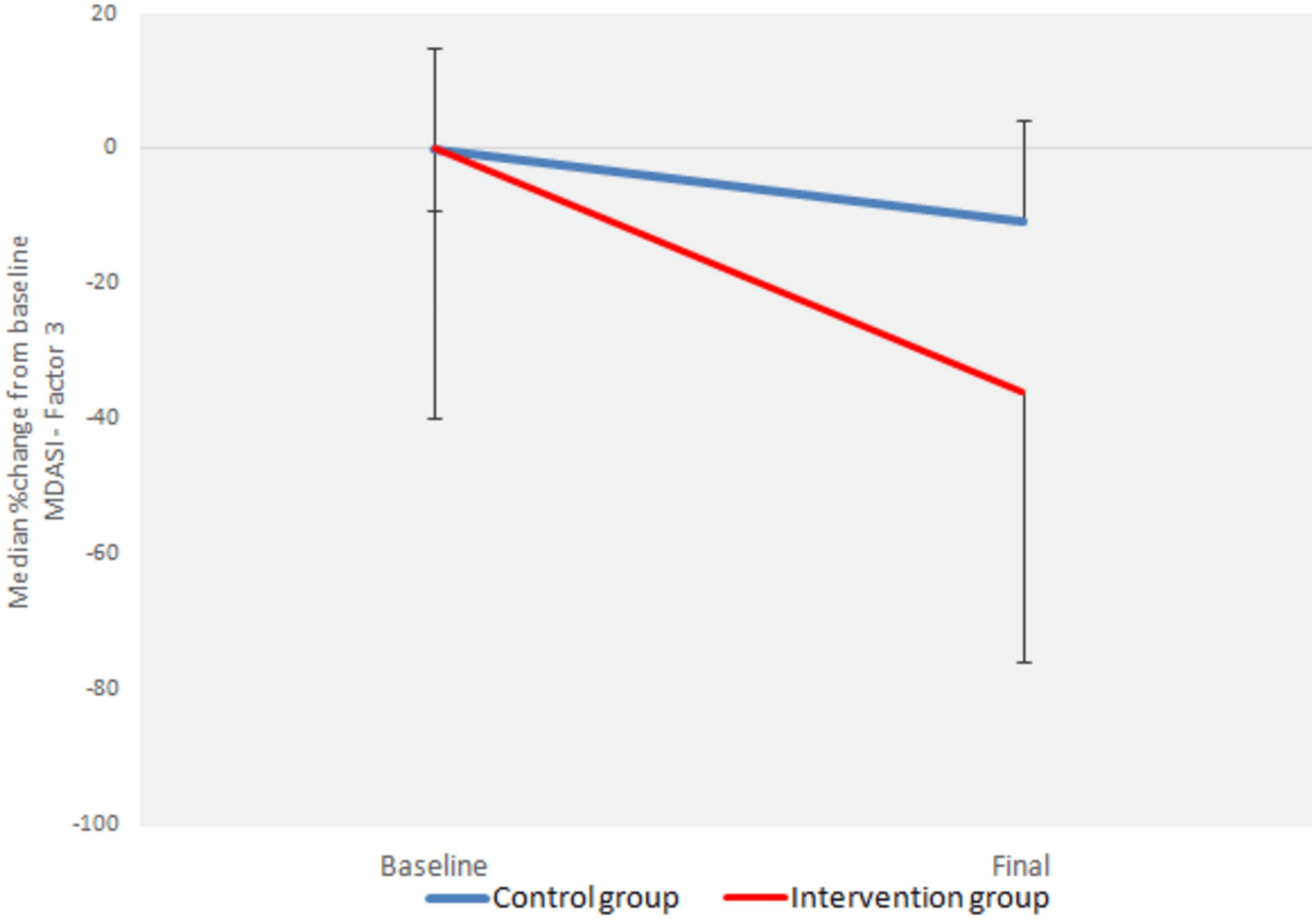
Median percentage change from baseline of MDASI Questionnaire - Factor 3 MDASI: M.D. Anderson Symptom Inventory.

There was a statistically significant interaction between the factors “group” and “time” (p < 0.0005) for all three variables of the MDASI questionnaire (Figures 1-3).

Moreover, there was a statistically significant reduction in the MDASI-Factor 1, MDASI-Factor 2, and MDASI-Factor 3 variables from baseline to the end of radiotherapy for both the control group (p < 0.0005) and the intervention group (p < 0.0005) (Figures 1-3).

There was a statistically significant difference between the groups at baseline (p < 0.0005 for MDASI-Factor 1, p = 0.004 for MDASI-Factor 3) and at the end of radiotherapy (p < 0.0005 for both MDASI-Factor 1 and MDASI-Factor 3) (Figures 1, 3).

There was no statistically significant difference between the groups for MDASI-Factor 2 at baseline (p = 0.153), but a significant difference was observed at the end of radiotherapy (p = 0.008) (Figure 2).

There was a statistically significant difference in the absolute change from baseline to the end of radiotherapy in all three variables, adjusted for the baseline value, between the groups (p < 0.0005) (Figures 1-3).

There was also a statistically significant difference in the percentage change from baseline to the end of radiotherapy in the MDASI-Factor 1 and MDASI-Factor 3 indices between the groups (p < 0.0005) (Figures 1, 3).

However, we did not compare the percentage change from baseline to the end of radiotherapy for the MDASI-Factor 2 index between the groups because many baseline values were rated as 0.

Information Styles Questionnaire (ISQ)

For the variable Information-Disease and Treatment, there was a statistically significant interaction between the factors “group” and “time,” F(1, 214) = 5.86 (p = 0.016). On the other hand, there was no statistically significant interaction for the variable Information-Psychological, F(1, 214) = 0.855 (p = 0.356).

There was a statistically significant difference in both Information-Disease and Treatment and Information-Psychological variables from baseline to the end of radiotherapy for both groups (p < 0.0005) (Table 4).

**Table 4:** Mixed-model ANOVA by two factors for the ISQ questionnaire variables SD: standard deviation, SE: standard error, BG: between group, WG: within group, ISQ:  Information Styles Questionnaire.

Variables	Group	Baseline	Final	p-value_WG_	Absolute Change Baseline-Final
		Mean ± SD	Mean ± SD		Adjusted Mean ± SE
Disease and treatment	Control	18.26 ± 2.94	18.46 ± 3.35	<0.0005	0.38 ± 0.24
	Intervention	16.58 ± 3.74	15.97 ± 3.83	<0.0005	-0.76 ± 0.22
	p-value_BG_	<0.0005	<0.0005		= 0.001
Psychological	Control	7.85 ± 1.73	8.46 ± 2.10	<0.0005	0.42 ± 0.13
	Intervention	7.84 ± 1.85	8.26 ± 1.95	= 0.002	0.61 ± 0.15
	p-value_BG_	= 0.974	= 0.477		= 0.336

For the variable Information-Disease and Treatment, there was a statistically significant difference between the groups at baseline (p < 0.0005) and at the end of radiotherapy (p < 0.0005), while for the variable Information-Psychological, there was no statistically significant difference between the groups either at baseline (p = 0.974) or at the end of radiotherapy (p = 0.477) (Table 4).

For the Information-Disease and Treatment variable, there was a statistically significant difference in the absolute change from baseline (0.38 ± 0.24) to the end (−0.76 ± 0.22) of radiotherapy, adjusted for the baseline value, between the groups (p = 0.001) (Table 4). However, for the Information-Psychological variable, there was no statistically significant difference in the absolute change from baseline (0.42 ± 0.13) to the end (0.61 ± 0.15) of radiotherapy, adjusted for the baseline value, between the groups (p = 0.336) (Table 4).

Regarding the percentage change in the Information-Disease and Treatment variable, there was a statistically significant difference from baseline (1.69 ± 13.32) to the end (−2.83 ± 13.04) of radiotherapy between the groups parametrically (p = 0.013) (Table 4). However, for the Information-Psychological variable, there was no statistically significant difference in the percentage change from baseline (8.92 ± 20.05) to the end (6.82 ± 18.44) of radiotherapy between the groups parametrically (p = 0.425) (Table 4).

## Discussion

To the best of our knowledge, the present study is the first scientific work in Greece that investigates the informational needs, fear, and symptoms associated with radiotherapy in women with breast cancer. In conjunction with the study by Ouzouni et al. [7], these represent the first findings in the Greek population.

Based on the results of this study, according to the mixed model ANOVA for the variable “Fear,” there was a statistically significant interaction between the factors “group” and “time” (p < 0.0005).

The analysis of the total fear score, as well as its subcategories, showed that the intervention group experienced improvement in fear. There was a statistically significant difference in the “Fear” index from the baseline to the end of radiotherapy in the intervention group (p < 0.0005). Fear levels improved in members of the intervention group due to the informational brochure leaflet.

Similar findings were reported in the study by Singer et al. [17], where a significant number of patients initially expressed strong fear of chemotherapy, followed by lymphadenectomy and radiotherapy. As time progressed, fear of treatments diminished, particularly in relation to radiotherapy, which showed an average decrease from a score of 2.7 at the start to 2.2 after completion of adjuvant therapy.

Previous research has indicated that the highest-ranked fear was associated with the possibility of undergoing radiotherapy [18]. This was also confirmed by our study, which demonstrated a statistically significant difference in the fear of radiation effectiveness index from baseline to the end of radiotherapy in both the control group (p < 0.0005) and the intervention group (p < 0.0005). Patients’ fear of radiation effectiveness was reduced at the end of radiotherapy in the intervention group.

Given that patients often have difficulty understanding the mechanisms of ionizing radiation for therapeutic purposes, fear of potential acute or chronic side effects of radiation therapy may sometimes exceed the fear of cancer itself. This can affect treatment compliance and even lead to failure to complete therapy [15]. Our study revealed similar findings, with a statistically significant difference in the fear index from baseline to the end of radiotherapy in both the control group (p < 0.0005) and the intervention group (p < 0.0005) for the factor “fear of illness during radiotherapy.” Conversely, cancer and its treatments can significantly alter patients’ daily activities, especially in cases of advanced disease [19]. These circumstances can significantly affect quality of life [19] and may eventually lead to mood disorders such as psychological distress, anxiety, and depression [20].

In the present study, fear related to the impact of radiotherapy on daily life decreased in the intervention group by the end of radiotherapy sessions. The informational brochure leaflet played an important role in this reduction. The diagnosis of cancer, along with subsequent treatments, can lead to heightened anxiety [21]. Contrary to our findings, Wallace et al. [22] evaluated the impact of different radiotherapy schedules on anxiety levels and found no notable differences between short and long regimens, either before or after radiotherapy, possibly due to the extended treatment schedule of six weeks.

Our primary findings revealed that the fear of side effects of radiotherapy and relationships with family and friends improved in the intervention group (p < 0.0005). Social isolation is a recognized risk factor for inadequate mental health in the wider community [7]. Similarly to our findings, in a previous study [7], individuals who received support during their radiotherapy exhibited higher scores in the factor “fear of side effects of radiotherapy and relationships with family and friends” compared to those who underwent the sessions independently (p = 0.01). Likewise, Gimson et al. [23] discovered that receiving emotional support from family and friends, whether in a hospital setting or at home, significantly contributed to reducing anxiety. Several participants noted a decrease in anxiety levels when family and friends assisted them with their hospital appointments. Conversely, supporting research indicates that cancer patients often have apprehensions about depending on caregivers [24]. This anxiety regarding dependency is one of the factors that contributes to suicidal ideation and worsens the psychological health of the patient [7].

Receiving a cancer diagnosis frequently signifies significant emotional, physical, and social distress; consequently, contemporary cancer treatment should include a variety of psychosocial interventions to enhance patients' overall quality of life [20]. Our study found through statistical analysis that confusion improved, and tension, depression, anger, fatigue, and vigor decreased after the end of radiotherapy in the intervention group. The control group also showed a small decrease in these mood states at the end of the sessions.

Although radiotherapy for breast cancer can have a very beneficial impact on prognosis or cure, it may also significantly increase levels of anxiety and depression. The findings of our study confirm previous research; specifically, Lewis et al. discovered that anxiety levels, measured using visual analogue scales (VAS), tend to be highest at the onset of radiotherapy, particularly during the simulation phase and the initial treatment sessions. Thereafter, these levels decline substantially [25].

However, contrary to our study, another study found that throughout radiotherapy, patients experienced peak levels of pain, sleep disturbances, fatigue, worry, and anxiety. Nonetheless, over 50% of patients continued to encounter sleep disturbances, fatigue, worry, and anxiety even after completing radiotherapy [26]. While undergoing radiotherapy, patients reported greater symptom-related distress.

Our study has shown that for all three MDASI factors-covering treatment-related symptoms-the intervention group significantly reduced symptom levels compared to the control group. This reduction resulted from a comprehensive approach and the provision of information. Similar to our results, clinical studies have found that patients with breast cancer experienced their highest levels of anxiety prior to starting radiotherapy, which then notably decreased as treatment advanced [27]. This pattern was observed in both somatic anxiety and state anxiety, aligning with findings from earlier research [25]. Studies indicate that many patients do not fully understand radiotherapy or its potential side effects (such as damage to internal organs, skin damage, pain, and fatigue) prior to treatment. Insufficient interaction with radiotherapy experts can also contribute to anxiety about unknown aspects of the process. Consequently, as radiotherapy continues, a notable reduction in anxiety levels reflects patient adjustment and may be associated with the support offered by the radiation therapy team once treatment begins [27].

On the other hand, contrasting our findings, previous research revealed a notable effect of symptoms experienced after radiotherapy on patients' quality of life (QoL), as well as a strong link between anxiety and QoL. The intensity of symptoms following radiotherapy was associated with diminished QoL and increased anxiety. Complications from symptoms such as fatigue, insomnia, pain, and diarrhea after radiation therapy were strongly linked to lower quality of life and increased anxiety. At the end of treatment, quality of life, energy levels, and daily functioning were lower compared to baseline [28].

Unfortunately, many patients lack awareness of what to expect during radiotherapy and may struggle to absorb the volume of information typically presented during consultations with a radiation oncologist [29].

For the Information-Disease and Treatment variable, patients in the intervention group, because of the informational brochure leaflet, required less information at the end of radiotherapy. Conversely, other researchers found that most patients reported minimal or no understanding of breast cancer radiotherapy, indicating that the information they received prior to their consultation with the radiation oncologist was insufficient [6]. Moreover, in the study by Matsuyama et al. [29], almost 80% of patients reported having little to no understanding of basic principles related to radiation therapy at their baseline appointment.

The findings from the survey by Halkett et al. [16] illustrated that patients undergoing radiotherapy possess diverse information needs extending beyond knowledge of their illness and treatment options, including logistical details related to radiotherapy. These findings were confirmed by our research, where patients expressed a greater need for information about disease and treatment, and a lesser need for information related to psychological aspects.

The use of an informational brochure leaflet has already shown success in previous studies [16]. Patients primarily sought written and verbal information sources, as these allowed them to meet specific informational needs as they arose [16].

The patients' attitude after receiving information plays a crucial role in the outcome of their illness. The sense of having control over the disease provides empowerment to manage it more effectively and facilitates adaptation [30].

Limitations

The current study has a number of limitations, even though it provides the experiences of 216 cancer patients who underwent radiation treatment. Despite its diversity, the study population may be compromised by socioeconomic and geographic factors, as it was restricted to patients from two hospitals. However, it boasts several strengths, notably being the first survey in Greece to address the fear of radiotherapy in women with breast cancer and their informational needs about therapy. Consequently, Greek healthcare workers in oncology departments can use this resource to identify, address, track, and potentially avert symptoms of fear, as well as to improve patient education, ultimately leading to better healthcare delivery.

## Conclusions

The analysis of the total fear score, as well as its subscales, revealed that patients in the intervention group showed improved levels of fear related to radiotherapy. Also, in all subcategories of the POMS questionnaire, which examines psychological moods, the intervention group outperformed the control group, thus reducing negative emotions. In the MDASI questionnaire, the intervention group significantly reduced symptoms in all three subscales compared to the control group. Finally, regarding information needs, breast cancer patients in both groups did not require additional information after the end of the intervention.

The findings of this study emphasize the widespread fears and misunderstandings surrounding breast cancer radiotherapy, showing that patients' real treatment experiences often exceed their initial expectations. The presented findings and the suggested strategies could help healthcare professionals, educators of radiation therapists, nurses in the field of radiotherapy, and cancer researchers to establish effective methods for patient education. Additional research is needed to investigate how these informational needs can be more effectively fulfilled.
